# Calciphylaxis in chronic kidney disease: a systematic review of pathomechanisms and risk factors for calcific uremic arteriolopathy

**DOI:** 10.1080/0886022X.2026.2710397

**Published:** 2026-08-10

**Authors:** Cecilie Søndergaard, Subagini Nagarajah, Jianlin Shen, Else-Marie Bladbjerg, Alexandra Scholze

**Affiliations:** a Department of Clinical Research, University of Southern Denmark, Odense, Denmark; b Department of Nephrology, Odense University Hospital, Odense, Denmark; c Department of Orthopedics, Putian University, Putian, China; d Unit for Thrombosis Research, Department of Clinical Biochemistry, University Hospital of Southern Denmark, Esbjerg, Denmark; e Department of Regional Health Research, University of Southern Denmark, Odense, Denmark; f Department of Cardiac, Thoracic, and Vascular Surgery, Odense University Hospital, Odense, Denmark

**Keywords:** CKD, hemodialysis, nephrogenic calciphylaxis, inflammation, vascular calcification

## Abstract

Calcific uremic arteriolopathy (CUA) is a rare, severe condition affecting patients with chronic kidney disease (CKD). The etiology of CUA is unclear. Here, we aimed to identify factors associated with its development. We reviewed the existing evidence on its pathomechanisms and risk factors. We searched EMBASE and PubMed up to February 2026, reviewing all studies that would facilitate a comparison of CKD patients with and without CUA. Of 2374 screened publications, 35 met our inclusion criteria. The factors that consistently emerged related to CUA were decreased albumin, higher alkaline phosphatase, diabetes, increased body mass index, female sex, vitamin K antagonist (VKA) use, increased calcium phosphate product, and calcification and thrombosis in subcutaneous small arteries. Factors that appeared to be likely associated with CUA were anemia, suboptimal dialysis efficiency, peritoneal dialysis, inflammation, arterial hypertension, smoking, and hepatobiliary disease. Findings on matrix Gla protein alterations implicated in mediating VKA effects, parathyroid hormone, and hypercoagulability were ambiguous in our literature analyses. In addition, we found that a standardized system for CUA skin biopsy evaluation is lacking. In summary, our review delineates the major risk factors and pathogenic parameters associated with CUA development. Furthermore, it underscores areas in which robust data on CUA pathomechanisms are lacking.

## 1. LAY SUMMARY

Calcific uremic arteriolopathy (CUA), also known as calciphylaxis, is a rare but serious condition that can occur in people with advanced kidney disease. It causes calcium to build up in small blood vessels, reducing blood flow and leading to painful skin wounds.

To better understand why CUA develops, we reviewed studies comparing people with chronic kidney disease who did and did not develop the condition. We found that several factors were commonly linked to a higher risk of CUA, including diabetes, higher body weight, low blood albumin levels, abnormal calcium and phosphate levels, use of certain blood-thinning medicines (vitamin K antagonists such as warfarin), and being female. Other possible risk factors included anemia, inflammation, smoking, high blood pressure, and less effective dialysis treatment.

Our findings suggest that CUA develops through a combination of factors affecting blood vessels, mineral balance, and overall health. More research is needed to understand exactly how these factors contribute to the disease and whether they can help identify patients at risk before symptoms appear. This could improve early detection, prevention, and treatment of this serious condition.

## 2. KEY MESSAGES

1. What is known: Calcific uremic arteriolopathy (CUA) is a rare, life-threatening complication of chronic kidney disease, but factors associated with its development have not previously been systematically and quantitatively compared between patients with and without CUA across the published literature.
2. This study adds: This systematic review identifies the factors most consistently associated with CUA development, including low albumin, elevated alkaline phosphatase, diabetes, higher body mass index, female sex, vitamin K antagonist use, and elevated calcium–phosphate product, and further highlights additional relevant factors, including smoking and suboptimal dialysis efficiency.
3. Potential impact: These findings may help improve early risk identification and guide future research into CUA mechanisms, prevention strategies, and standardized pathological assessment.

## 3. SOCIAL MEDIA ‘HOOK’

What drives calcific uremic arteriolopathy? Our systematic review identifies the factors most consistently associated with this rare complication of chronic kidney disease and highlights additional emerging risk factors.

## GRAPHICAL ABSTRACT

**Figure:**
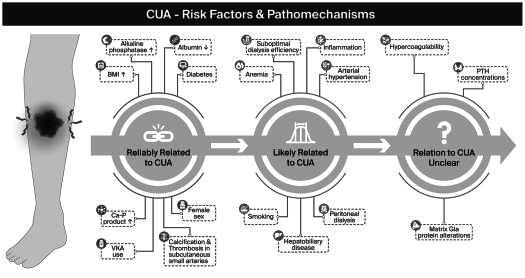

## 1. Introduction

Calcific uremic arteriolopathy (CUA), also termed calciphylaxis in chronic kidney disease (CKD) or nephrogenic calciphylaxis, is an infrequent condition that can develop in patients with kidney dysfunction. Affected patients are found to have indurated plaques of the skin or necrotic skin lesions that frequently fail to heal. Mortality is high, with a reported survival of approximately 50% at 6 to 12 months after diagnosis [1–3]. The pathogenesis likely involves reduced arteriolar blood flow in the skin, as evidenced by vascular calcifications and thrombosis, but is generally poorly understood [1,4]. While there is currently no evidence-based treatment for CUA, recently suggested approaches target the vascular calcifications [5,6] and tissue hypoxia [7,8].

CUA typically develops in advanced CKD [9]. Its onset is difficult to predict, as most of the hitherto described pathomechanisms, including cutaneous vascular calcification [10], are common in this population. Nevertheless, only a minority of at-risk patients develop the disorder. Improved understanding of CUA risk factors and pathomechanisms is a prerequisite for the development of early detection algorithms and new treatment modalities. A major challenge in CUA research is delineating the specific mechanisms and parameter combinations that give rise to this condition, as many of these factors may be intrinsic to CKD, particularly in its advanced stages. For this purpose, we exclusively selected and analyzed information that enabled the comparison of a CUA population with a suitable CKD control population.

This review aims to compare pathogenic parameters between CUA patients and CKD controls, quantify the reliability of these parameters and risk factors, and identify knowledge gaps warranting further investigation.

## 2. Methods

### 2.1. Literature search

We followed the PRISMA guideline for systematic reviews (Supplementary material, PRISMA checklist). The protocol was prospectively registered in PROSPERO on October 16, 2023, registration number CRD42023473048. The literature search was conducted in October 2023 after protocol registration, using the PubMed and EMBASE databases (Supplementary material, Search strings PubMed and EMBASE). A final database re-screening was done in February 2026. The searched literature covered the period from January 1, 1963, to February 09, 2026.

### 2.2. Study selection

Two reviewers (CS, AS) independently screened article titles and abstracts using the Covidence software for managing systematic reviews. Only publications with full texts were considered. Identified studies were assessed for eligibility by applying predefined inclusion and exclusion criteria according to the PROSPERO protocol. Both reviewers were blinded to each other’s choices, and discrepancies were later resolved by consensus. Inclusion criteria: Study patients with CKD and calciphylaxis; study population in the majority adults (≥18 years); only studies for which a comparison of calciphylaxis cases to a respective CKD control group could be performed. This meant a) the control group was from the same publication or named in the publication, with *n* ≥ 5 participants in the control and calciphylaxis group; b) for large cohort studies with *n* ≥ 100 calciphylaxis cases, the control group could be obtained from a published or publicly available database with respective national CKD control cases. Control patients were required to match calcific uremic arteriolopathy (CUA) cases at least by presence or absence of renal replacement therapy and/or CKD stages. Controls from the literature should additionally be matched by country/region of residence. Exclusion criterion: Studies on patients with functioning kidney transplant. Articles were included if they reported any kind of intervention, exposure, risk factor, or pathomechanism that may be linked to CUA pathogenesis, and we did not implement restrictions on the publication period or language.

### 2.3. Quality assessment

We used the National Institute of Health Quality Assessment Tool to evaluate the internal validity of selected studies. This method of quality assessment considers the study design of each assessed article, which was rated from good to poor.

### 2.4. Data extraction and synthesis

Data extraction was conducted independently by CS and AS using a custom-made Excel document (https://osf.io/mc36k/files/osfstorage; Systematic review CUA vs matched control data.xlsx). Extracted data were compared and discrepancies resolved by consensus. We retrieved any reported interventions, exposures, risk factors, and pathomechanisms for CUA cases and their respective controls to investigate CUA pathogenesis. Matched parameters were not compared. Based on the rare nature of CUA, the studies often include small numbers of cases. Therefore, we choose an inclusive approach with respect to study design and outcome reporting. The resulting diversity precluded meta-analysis, which was defined *a priori* in the PROSPERO protocol. We followed the SWiM guideline for synthesis without meta-analysis [11] and tabulated all studies. We also present votes for reported parameters based on effect direction (lower, equal, or higher) in CUA cases. In our literature analyses, we used vote counting according to the Cochrane Handbook for Systematic Reviews of Interventions [12], with votes indicating the direction of effects for parameters compared between CUA cases and their control groups, without considering statistical significance or effect size [12]. For our analyses, we used the exact value, including its decimal places, as originally reported for each parameter. When only an odds ratio or hazard ratio for a parameter was reported, we used the ratio’s effect direction as the vote. For vote counting, only parameters that were reported in at least two different publications were considered, and for parameters reported multiple times in a single publication, e.g. at CUA diagnosis and 6 months prior to CUA diagnosis, we used only one vote because we did not regard these data as independent from each other. This approach was supported by the fact that the respective “related” votes in all studies showed the same effect direction.

During our analyses, we performed the following calculations using Microsoft Excel 2013 (Microsoft Corporation): percentages of female participants [13], cases of peritoneal dialysis (PD) [14], and cases of diabetic nephropathy [15]; mean and standard deviation of tissue gadolinium concentration [16]; and medians and interquartile ranges of all parameters used from the Dialysis Outcomes and Practice Patterns Study (DOPPS) database.

## 3. Results

### 3.1. Literature screened

**Figure 1.**
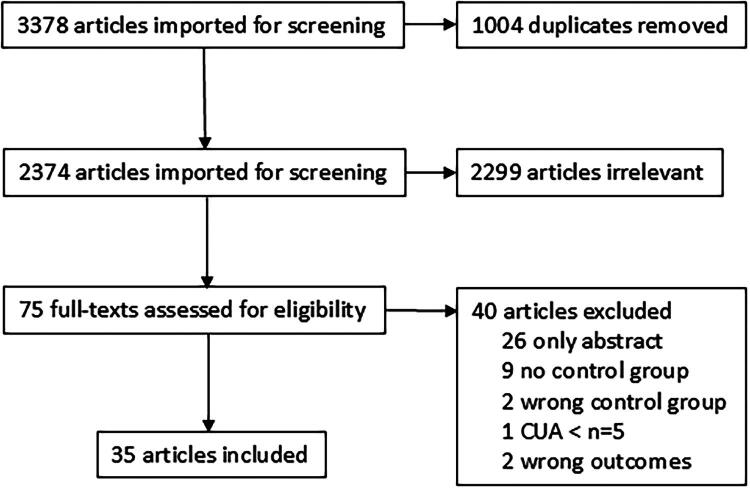
Screening and selection process for the review. CUA—patient population with calcific uremic arteriolopathy.

Overall, 3378 articles were identified, of which 2374 were selected for screening of abstracts, and therefrom 75 full texts were screening. Finally, we included 35 articles in our review (Figure 1).

### 3.2. Study characteristics

The articles span 1990 to 2025. We analyzed the geographical origin of published studies. This review identified information on CUA-related parameters from four continents (North America, Europe, Asia, and Oceania). The majority of studies were performed in North America and Europe. No publications from South America and Africa were available.

### 3.3. Findings from our quality assessment

All articles underwent quality assessment (Supplementary Table S1). We used the National Institutes of Health Study Quality Assessment Tool. This tool provides a structured, transparent, and study design-specific approach for evaluating methodological quality and risk of bias across different types of study designs. This was the case in the present systematic review, as we had prespecified an inclusive approach in the systematic review protocol. During our final analysis, we extracted data from case-control, cross-sectional, and observational cohort studies as well as one RCT. As a result, 60% of articles were rated ‘Good’, 29% ‘Fair to good’, and 11% ‘Fair’; all research was deemed proficient. Most articles did not include a sample size justification or it was not applicable. Among the articles rated ‘Good’, descriptions of the blinding of assessors were sometimes missing. Articles rated as ‘Fair to good’ mainly lacked descriptions of potential confounding variables, subcategories had not been matched, or the background population was not clearly specified. Articles rated ‘Fair’ mostly did not adjust for confounding variables or lacked specific descriptions of the control or CUA groups.

### 3.4. Definitions of the CUA diagnosis

As the method of diagnosing CUA is not yet standardized [1], we analyzed the definitions of CUA that were used in the identified studies. Supplementary Table S1 provides diagnostic approaches that were reported by different authors. We found no two different research centers that used the exact same criteria for definition. Three articles used biopsy-proven CUA only, one article used radiographic criteria only, four articles used treating physicians’ diagnosis without further definition, eight publications reported use of clinical criteria alone, three articles stated the use of Hayashi’s criteria or criteria in line with Hayashi’s criteria, and sixteen articles reported combinations of different clinical and histopathological criteria (Supplementary Table S1).

### 3.5. Frequency of risk factors and pathogenic parameters in CUA

#### 3.5.1. Demography

**Figure 2.**
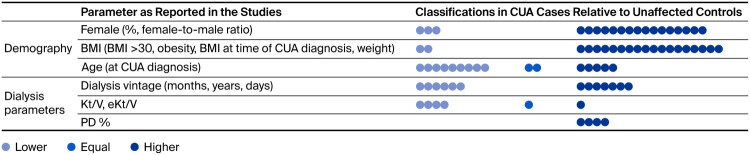
Vote counting of demographic and dialysis parameters in CUA that were investigated in at least two studies with a suitable CKD control population. The effect direction for the comparison of CUA to control is classified as lower (light blue), equal (azure blue), or higher (dark blue). The number of circles represents the number of studies. BMI; body mass index, CUA; Calcific Uremic Arteriolopathy, eKt/V; Equilibrated Kt/V (Dialysis Dose), Kt/V; Dialysis Adequacy Measurement, PD; Peritoneal Dialysis.

Related parameters were categorized into domains, although there is overlap between them. Most CUA populations consisted of more females than males, and a higher BMI or obesity was more prevalent among CUA patients than controls. The CUA population tended to be younger than the controls, but there was no clear predominance (Figure 2).

#### 3.5.2. Dialysis parameters

Regarding dialysis-related characteristics, dialysis vintage was comparable between CUA and non-CUA patients. Hemodialysis treatment adequacy, as measured by Kt/V, was more often lower in CUA, and the fraction of patients with PD treatment was higher [17] across all studies reporting this parameter (Figure 2). Several parameters of dialysis implementation were sparsely investigated, such as dialysate calcium concentration, HDF use, citrate dialysis, and catheter access, as was information on the dialysis population, such as HD initiation before PD, late patient referral to dialysis, or serum bicarbonate concentration.

#### 3.5.3. Bone and calcium-phosphate metabolism and calcification processes

**Figure 3.**
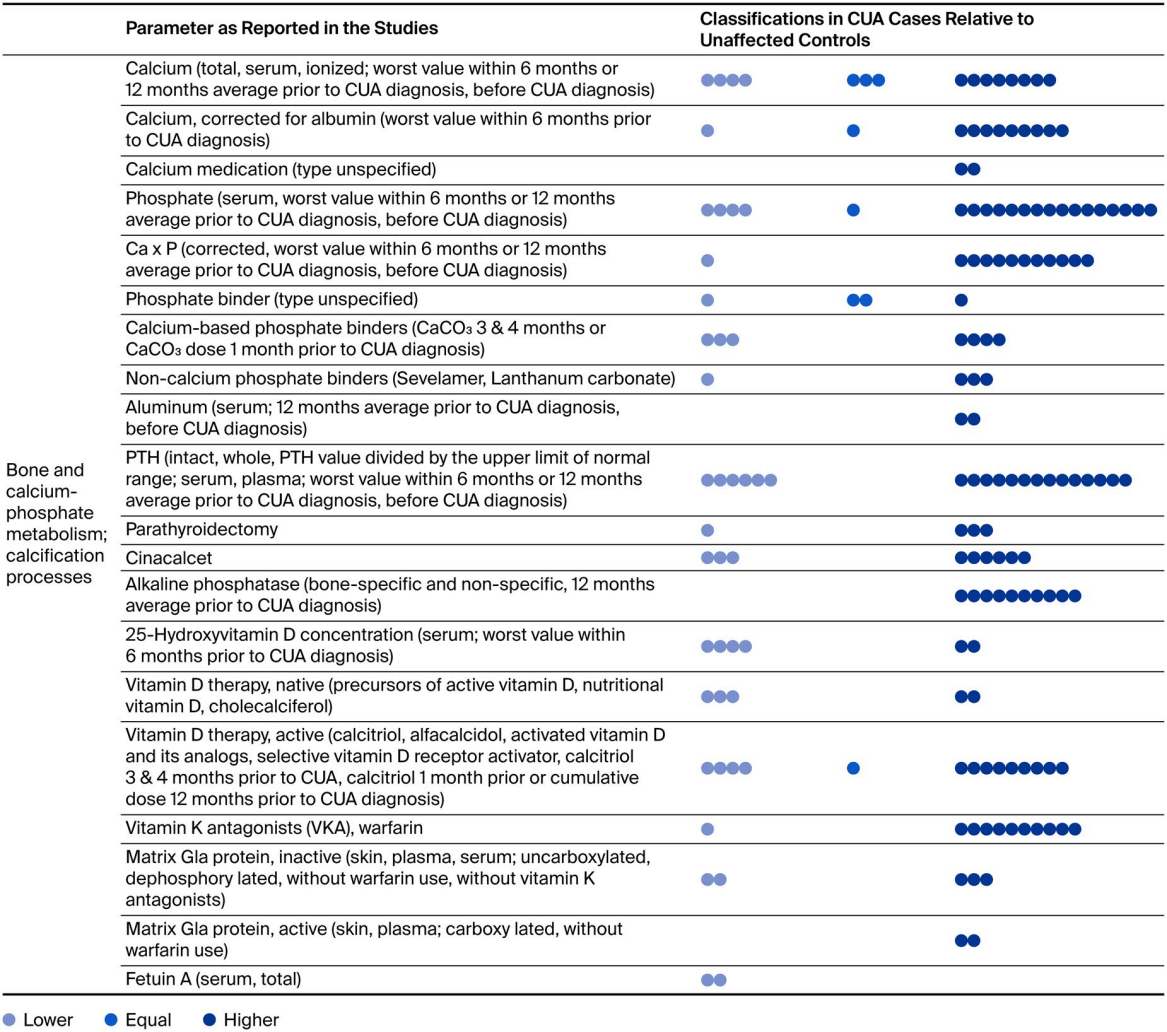
Vote counting on parameters of bone, calcium phosphate metabolism, and calcification processes in CUA that were investigated in at least two studies with a suitable CKD control population. The effect direction for the comparison of CUA to control is classified as lower (light blue), equal (azure blue), or higher (dark blue). The number of circles represents the number of studies. Ca x P; Calcium-Phosphorus Product, CaCO3; Calcium Carbonate, CUA; Calcific Uremic Arteriolopathy, PTH; Parathyroid Hormone, VKA; Vitamin K Antagonist.

Most studies investigated parameters related to calcium phosphate metabolism or calcification processes. Calcium concentrations in CUA were nearly as often lower or equal [18] as they were higher in CUA compared with the controls [13,19]. Only albumin-corrected calcium was regarded as higher in CUA cases in most of the studies. The phosphate concentration was nominally higher in CUA cases in most of the studies. We found that the studies reported a nominally higher calcium phosphate product in CUA in all but one of the studies that had investigated this parameter (Figure 3).

The frequency of phosphate binder therapy with calcium-based formulations was comparable between CUA cases and controls. Non-calcium phosphate binders were more frequent in CUA cases [20,21]. Among studies reporting on PTH, concentrations in CUA were higher in around 70% of studies, while the frequency of parathyroidectomy [22,23] and the use of cinacalcet [24,25] were generally higher in CUA populations.

Remarkably, all 10 publications that reported any alkaline phosphatase showed higher concentrations in CUA [15,26]. Serum 25-hydroxyvitamin D was somewhat more frequently lower in CUA. Reports on therapy with native vitamin D were rather sparse and overall comparable between CUA patients and controls (Figure 3). Frequency of medication with vitamin D receptor activating formulations was higher in CUA [27] in around 65% of 14 reporting studies.

Concentrations of vitamin D-binding protein, 1,25-dihydroxyvitamin D, or fibroblast growth factor 23 (FGF23) were only investigated in one study each.

Except for Vitamin K Antagonist (VKA) therapy, additional parameters related to vascular calcification were seldom compared. Nearly all studies that analyzed VKA reported a higher frequency of VKA use in CUA [28], and five studies investigated the inactive form of the calcium-binding matrix Gla protein (MGP). Lower and higher MGP concentrations were reported with comparable frequency, while the two studies that also investigated the vitamin K-dependently activated form of MGP both reported higher amounts in CUA [29–33]. The liver-derived fetuin A protein was twice investigated, with both studies showing lower concentrations in CUA [19,33]. The fraction of serum fetuin A contained in calciprotein particles (CPPs), the transition time from primary to secondary CPPs (T50), and magnesium concentrations were not compared in more than one study each to date.

#### 3.5.4. Inflammation and nutrition

**Figure 4.**
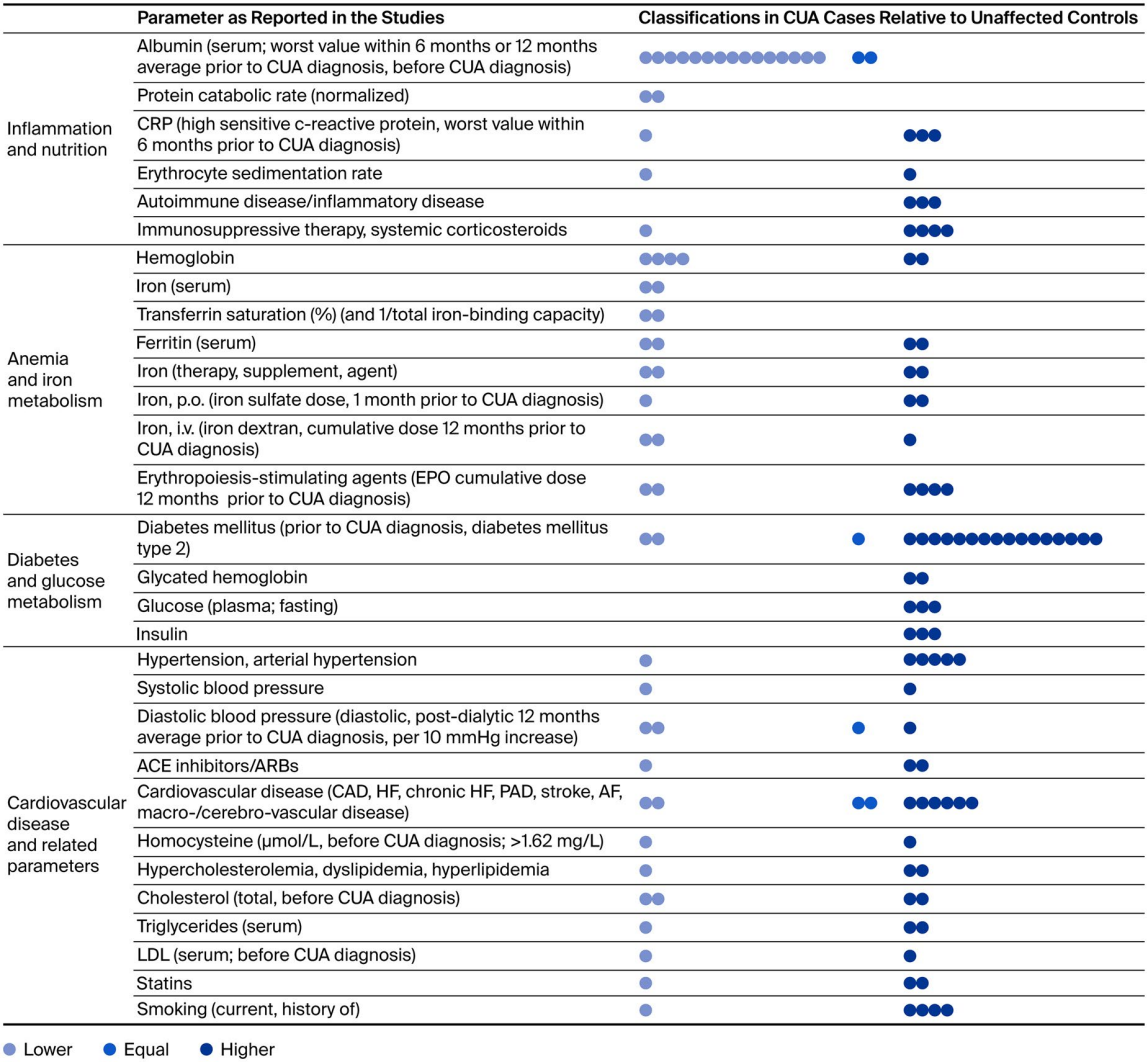
Vote counting on parameters of inflammation and nutrition, anemia and iron metabolism, diabetes and glucose metabolism, and cardiovascular disease-related parameters in CUA that were investigated in at least two studies with a suitable CKD control population. The effect direction for the comparison of CUA to control is classified as lower (light blue), equal (azure blue), or higher (dark blue). The number of circles represents the number of studies. ACE; Angiotensin-Converting Enzyme, ARBs; Angiotensin II Receptor Blockers, CAD; Coronary Artery Disease, CRP; C-Reactive Protein, CUA; Calcific Uremic Arteriolopathy, EPO; Erythropoietin, HF; Heart Failure, LDL; Low-Density Lipoprotein, PAD; Peripheral Artery Disease.

Parameters of inflammation and nutrition were rarely investigated except for serum albumin. For albumin, out of 16 studies, none reported higher concentrations in CUA, with the majority reporting lower albumin [34–36]. Two studies reported a lower normalized protein catabolic rate in CUA [35,37]. Comparison of the erythrocyte sedimentation rate between CUA and controls did not point in one direction, but CRP was more often higher in CUA [23]. Autoimmune and inflammatory diseases were more frequent in patients with CUA [38,39], as was the prescription of immunosuppressive therapy, including systemic steroids (Figure 4).

#### 3.5.5. Anemia and iron metabolism

Aspects of anemia were also compared; however, they were assessed less frequently than calcium phosphate metabolism parameters (Figure 4). Hemoglobin was more often lower in CUA, and the same was true for serum iron concentration and iron bound to transport proteins [21,25]. The use of erythropoiesis-stimulating agents in CUA cases was more frequent [36]. For iron therapy and ferritin concentrations, the votes were evenly distributed between CUA cases and controls.

#### 3.5.6. Diabetes and glucose metabolism

Most studies reported a higher prevalence of diabetes in the CUA population [40,41], higher concentrations of glycated hemoglobin, higher glucose, and greater frequency of insulin prescription [15].

#### 3.5.7. Cardio-vascular disease and related parameters

The relation between CUA and classical cardiovascular disease and cardiovascular risk factors is less well-characterized. Smoking and a diagnosis of arterial hypertension were more frequent in CUA [3,22] and a combined vote for cardiovascular disease was slightly more frequent [39]. For actual blood pressure (systolic, diastolic), homocysteine, total and LDL cholesterol, dyslipidemia, and triglycerides, no predominance of vote direction was observed. The same applied for the use of angiotensin-converting enzyme inhibitors, angiotensin II receptor blockers, and statins (Figure 4).

#### 3.5.8. Coagulation and related parameters

**Figure 5.**
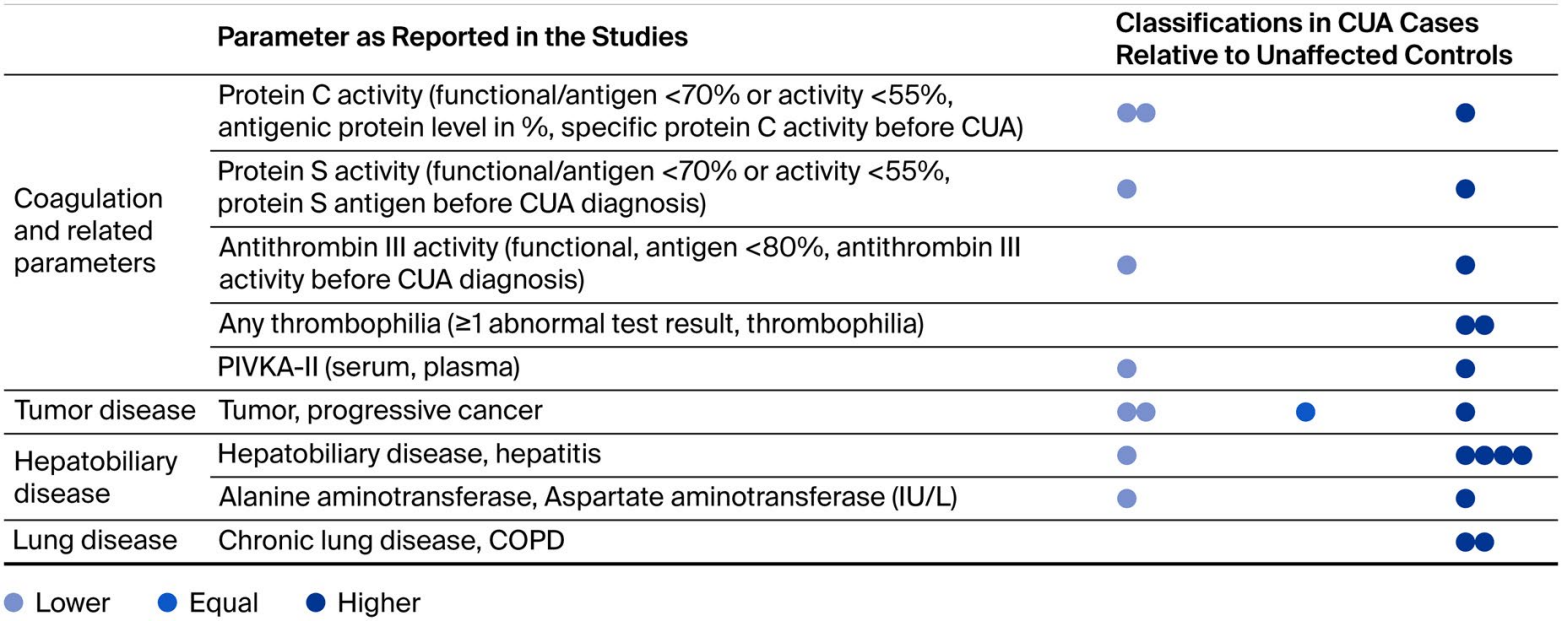
Vote counting on parameters of coagulation and comorbidities in CUA that were investigated in at least two studies with a suitable CKD control population. The effect direction for the comparison of CUA to control is classified as lower (light blue), equal (azure blue), or higher (dark blue). The number of circles represents the number of studies. CUA; Calcific Uremic Arteriolopathy, EPO; PIVKA-II; Protein induced by vitamin K absence or antagonist II.

Coagulation disturbances have been suggested to contribute to CUA pathogenesis. Nevertheless, we identified only sparse coverage of the topic (Figure 5).

Two studies reported a higher frequency of any thrombophilia [27,41], while for protein C, S, and antithrombin III activity [38,41,42], as well as protein induced by vitamin K absence or antagonist II (PIVKA-II) [30,32], no direction of vote dominated. Other parameters were sparsely compared, including platelet count, aspirin use, international normalized ratio (INR) without VKA use, deep vein thrombosis, and lupus anticoagulant.

#### 3.5.9. Additional comorbidities

Regarding comorbidities, studies investigating hepatobiliary disease reported a higher frequency in CUA, while no clear tendency for tumor disease was observed. Chronic lung disease was more frequent in CUA (Figure 5), and other conditions, such as recurrent severe hypotensive episodes [37] or transcutaneous pO2 analysis [14], were only compared once. Only one study compared single-nucleotide polymorphisms, for example, for the adenosine-producing 5′-nucleotidase CD73 and the vitamin D receptor, between CUA and a control population [43].

#### 3.5.10. Comparison of votes from different continents

This review provides cross-regional information on CUA-related parameters from 4 continents (Supplementary Table S2). We selected all parameters, for which at least one vote per continent was available. The majority of votes originated from North America and Europe. Votes from Asia originated from three, and votes from Oceania from two studies. Based on this limited data availability, we did not count the votes but describe an overall impression. A higher frequency of females in the CUA population seemed to apply to North America, Europe, and Oceania, while for Asia this was not clear. A higher BMI, lower albumin concentration, and higher frequency of diabetes in CUA were reported from all 4 continents. For calcium and PTH, higher concentrations in CUA seemed to dominate in this comparison between continents. For the remaining parameters, the data were too sparse and too heterogeneous to be compared (Supplementary Table S2).

#### 3.5.11. Information derived from skin biopsies

We analyzed information from histological comparisons of skin biopsies between CUA patients and matched CKD controls to gain insight into pathophysiological processes. Analyses of skin biopsies can provide pathomechanistic insights through disease-related cellular, structural and molecular changes. Advanced methods allow for analysis of immune pathways and signal transduction mechanisms. Unfortunately, although meaningful analysis methods were reported, it was not possible to apply vote counting for distinct parameters because a uniform description of items among the studies was lacking. For example, calcifications were described in relation to intravascular or extravascular structures, the depth of the skin layer, vessel size, or calcification morphology. Additionally, multiple parameters were compared in just one study.

Overall, cutaneous and subcutaneous calcifications, especially calcifications of small-sized arteries and arterioles, were described as more frequent and/or more extensive in CUA [44,45]. Two publications described calcifications in CUA as circumferential [26,28]. Moreover, studies repeatedly showed a higher frequency of thrombosis in CUA, especially in small arteries and small vessels (Supplementary Table S3). Results on perieccrine calcifications varied across studies. Medial calcification and intimal fibroplasia were frequently named as inclusion criteria, but we did not identify publications that substantiated this in a direct comparison of CUA cases to suitable controls. In contrast, one study described medial calcinosis of Mönckeberg type as the typical form in end-stage kidney disease (ESKD) without CUA [28]. Another study did not identify a higher frequency of intimal hyperplasia in CUA skin biopsies compared with amputation samples from patients with ESKD [44]. Other parameters were compared only once, including osteogenic processes, deposition of metals [16], frequency and location of angioplasia [46], and extracutaneous calcifications [45] (Supplementary Table S3).

## 4. Discussion

This review identified 35 studies that allowed for comparison of patients with CUA with a suitable CKD control population, providing clues about CUA risk factors and pathomechanisms. We classified factors as consistently related to CUA, if a reasonable number of studies had investigated this factor and the vote in the clear majority of studies showed the same direction. If a smaller number of studies had investigated a factor and most of the votes were consistent, we classified this factor as likely associated with CUA. Furthermore, we report that the evidence for direct involvement of parathyroid hormone, for matrix Gla protein alterations mediating VKA effects, and for hypercoagulability as contributors to CUA pathogenesis was ambiguous.

In the reviewed literature, higher age was not a major risk factor for CUA, while female sex and obesity clearly were, in line with earlier reviews, as was diabetes [47–50]. Experimental data suggest that in women, peak oxygen uptake from blood during exercise is lower than in men [51], while skin perfusion models suggest lower inner skin blood perfusion in women [52]. Furthermore, patients with diabetes showed reduced transcutaneous oxygen tension [53]. In accordance with these findings, signs of subacute skin hypoxia that may precede acute ischemic necrosis in CUA were investigated. A reduction in transcutaneous pO2 in affected and unaffected body regions, and a presumably reactive increase in skin vessel proliferation, were reported. Both findings strongly suggest that mechanisms of chronic hypoxia require further investigation in CUA.

As most patients who develop calciphylaxis have ESKD [54], factors related to CKD severity must be highly relevant for CUA development. Among the modus operandi parameters of dialysis, our analyses indicate that lower dialysis adequacy and PD therapy may play a role in CUA pathogenesis. However, our findings from the literature cannot prove causality and may reflect patient characteristics. Interestingly, one investigation showed that a higher percentage of patients with CUA had experienced recurrent hypotensive episodes and had shifted from HD to PD treatment [37].

One of the most striking findings of our review is the overtly high frequency of hypoalbuminemia, which also precedes CUA development, as well as the lower albumin concentration in CUA compared with matched CKD controls in around 90% of reporting studies. A higher prevalence of hepatobiliary disease and inflammation in CUA can contribute to these lower albumin concentrations, as was reported in CKD [55]. Albumin also serves as a nutritional and protein-energy wasting marker [4,56], underlining its relevance in CUA. Increasing albumin to improve peripheral tissue hypoperfusion has been suggested [57] as has a lower mortality among hemodialysis patients who achieved higher albumin concentrations and dialysis adequacy [58]. Despite its well-known association with inflammation, the role of hypoalbuminemia in the pathogenesis of CUA remains unclear. Both the genesis of hypoalbuminemia and its consequences for CUA development require more attention, as does its potential to serve as an early warning sign.

Currently, most research on CUA is focused on calcium phosphate metabolism and calcification processes. Increased serum calcium concentrations compared with non-CUA cases, although not necessarily in relation to the normal range, were approximately as frequent in our review as equal and lower concentrations. This observation has previously been discussed [59]. Alternatively, albumin-corrected calcium is frequently reported. However, since albumin correction does not accurately reflect ionized calcium concentrations [60], and low albumin appears to be an inherent feature of CUA, analysis of ionized calcium would be preferable.

Nevertheless, our analyses leave no doubt about the involvement of both calcium and phosphate in the CUA pathogenesis, a fact particularly evidenced by the higher calcium phosphate product, also preceding CUA diagnosis, and the higher frequency of cutaneous and subcutaneous calcifications in CUA skin biopsies. Therefore, future research should focus on the conditions that promote calcification, especially in small arteries and skin structures in CUA. Interesting candidates were suggested, including upregulation of osteogenic processes in the skin and inflammation-related straightening of elastic fibers that might promote calcification [28,31].

The role of phosphate binders, both with and without calcium, cannot be deduced from our literature analysis. Their use frequency seems mainly to reflect CUA patients’ calcium phosphate status and treating physicians’ awareness thereof. Furthermore, our analysis did not support an independent role of PTH in CUA, as nearly one-third of studies report lower, not higher, PTH concentrations in CUA cases. PTH is part of complex disturbances of calcium, phosphate, and additional parameters that contribute to CUA. It was highlighted in earlier publications that therapeutic oversuppression of hyperparathyroidism might often be the underlying reason for low PTH levels prior to CUA development [25]. Therefore, it seems most promising to concentrate on the parameters that mediate PTH effects in CUA.

It has been suggested that patients with CKD and hemodialysis therapy, should obtain a PTH concentration of circa 2 to 9 times the upper normal for the respective assay. For non-dialysis patients, although the optimal PTH level is not known, it was suggested that treatment options should be considered when PTH concentrations progressively rise or are persistently above the upper normal for the respective assay [61]. For the prevention of CUA, it was suggested that PTH concentrations are maintained between 150 and 300 ng/mL [62]. Recommended treatment options include targeting modifiable factors, like hyperphosphatemia and high phosphate intake [61]. Specifically, for CUA prevention, the calcimimetic cinacalcet was suggested by some authors instead of activated vitamin D to avoid increasing serum calcium and phosphate concentrations [62,63].

The higher frequencies of parathyroidectomy, cinacalcet, and active vitamin D use observed in CUA most likely reflect prevailing therapeutic strategies for hyperparathyroidism. This does not exclude an additional risk exerted by oversuppression of PTH or by native or active vitamin D preparations when they result in higher calcium concentrations [64,65] or as direct result of active vitamin D use in uremia [66]. Lower concentrations of 25-hydroxyvitamin D, found in two-thirds of the investigated studies, can be interpreted as the result of the high diabetes frequency and increased body mass index in the CUA group [67,68], and the preferential prescription of active vitamin D.

To the most important findings of this review belongs the predominance of higher alkaline phosphatase (AP) concentrations in CUA cases, independent of the isozyme type investigated. Higher AP concentrations may reflect high bone turnover and soft-tissue calcification [69], increased levels of inflammation [70], as well as a higher production of reactive oxygen species in patients with CKD [71,72], which leads to increased AP through activation of the nuclear factor erythroid-derived 2-like 2 (Nrf2) [73,74]. Therefore, the contribution of high AP to CUA development, e.g. through pyrophosphate degradation, requires investigation.

A medication with VKA is widely seen as a risk factor for CUA [75], which is supported by our analysis. Nevertheless, we see a discrepancy between the consistently reported higher frequency of VKA use and the twice-reported higher amount of the active MGP. One possible explanation is that a higher level of MGP in patients with CUA is still too low relative to pro-calcification factors. It is also possible that VKAs contribute to CUA through a different pathomechanism. Warfarin was shown to induce ferroptosis [76], which may be worth investigating in CUA. Overall, the laboratory methods for both the determination of active and inactive MGP were very heterogenous with respect to employed antibodies, enrichment, and quantification methods.

We did not find clear support for the contribution of hypercoagulable states to CUA. So far, only a few studies have compared antithrombin III, protein S, and protein C in CUA cases with suitable controls. Our analyses showed that the results often were difficult to interpret due to concurrent VKA use. Moreover, the parameters investigated are mainly risk factors for venous thrombus formation. We suggest that future studies should include analyses on the role of the tissue factor pathway.

Finally, solid and comparable data from skin biopsies could profoundly support the understanding of CUA pathomechanisms. Our analyses showed that a uniform description of histopathological findings in CUA was lacking. We think that adopting a more uniform way of describing histopathology in CUA and performance of advanced analyses such as immunostaining, spatial gene-expression profiling, and spatial proteomics will improve understanding of CUA biology.

The role of skin biopsy in the diagnosis of CUA is still incompletely defined. Several authors emphasize that CUA is primarily a clinical diagnosis [9,77], though other authors do not agree [78]. The identification of specific histopathological features is challenging because skin samples from CKD patients with and without CUA, as well as from affected and unaffected skin areas, were reported to yield similar dermatopathological findings [10,79]. Nevertheless, skin biopsy may aid in differential diagnosis, particularly in atypical clinical presentations or when features overlap with other dermatologic conditions [4,80]. Uncertainty also persists regarding the optimal biopsy technique. Although there is consensus that biopsies should be sufficiently deep to include subcutaneous tissue, various approaches have been described, including punch biopsies, image-guided percutaneous core-needle biopsies, and deep full-thickness biopsies [81–83]. Further data on biopsy techniques, diagnostic yield, and short- and long-term complications are needed.

Our review found that skin biopsy findings were lacking unified evaluation items, which prevented accurate comparison of pathological features. We suggest that a uniform description of histopathological findings in CUA should be discussed between CUA researchers and adopted to enable comparability. A standardized scoring framework should include information on dermal ulceration, subcutaneous arterial calcification, intima hyperplasia and thrombi, as well as extravascular calcifications.

Although this was prespecified in our PROSPERO protocol, it is a limitation that our data analysis approach is descriptive and based on observational studies. Prospective studies, with the exception of one RCT, were not available, and the available data precluded meta-analysis. Therefore, causality cannot be proven, and preferred treatment patterns confound the parameters investigated [12]. CUA diagnostic criteria are currently inconsistent and identified studies were heterogenous in respect to study design, which reduces the comparability of findings but permitted incorporation of a wider evidence base. As data availability from different populations is currently insufficient, population representativeness needs to be addressed by future international multicenter CUA research collaborations.

The CUA-relevant parameters identified in our review cannot be directly used for prevention or treatment recommendations; however, they should be evaluated for their potential in CUA prevention, early detection, and treatment in appropriately designed clinical trials. This would include optimizing dialysis efficiency; managing hypertension, anemia, and diabetes; cautious weight reduction if overweight; smoking cessation; avoidance of hepatotoxic substances; replacement of VKAs with alternative anticoagulants when possible; and optimization of the calcium-phosphate product.

## 5. Conclusions

In conclusion, our review identified parameters that appear, consistently, i.e. decreased albumin, higher alkaline phosphatase, diabetes, increased body mass index, female sex, VKA use, increased calcium phosphate product, and calcification and thrombosis in subcutaneous small arteries, or likely, i.e. anemia, suboptimal dialysis efficiency, peritoneal dialysis, inflammation, arterial hypertension, smoking, and hepatobiliary disease, related to CUA pathogenesis. Future research is needed to uncover the exact mechanisms relating them to CUA development. Furthermore, their potential to serve as early warning signs should be investigated. We also outline those areas where more data is warranted. Finally, we explain the need to develop a common system to describe and evaluate CUA skin pathology to promote inter-institutional comparability of data and research findings.

## Supplementary Material

Supplementary material CUA.docx

## Data Availability

The data that support the findings of this study are openly available in the Open Science Framework (OSF): at https://doi.org/10.17605/OSF.IO/MC36K.
