# Supplementary material for: Calciphylaxis in chronic kidney disease: a systematic review of pathomechanisms and risk factors for calcific uremic arteriolopathy
Source: Ren Fail. 2026 Aug 10;48(1):2710397. doi: 10.1080/0886022X.2026.2710397 (PMC13459315; doi:10.1080/0886022X.2026.2710397)
Supplement: Supplementary file 1 [file IRNF_A_2710397_SM5290.docx]

**Supplementary material**

**Calciphylaxis in Chronic Kidney Disease: A Systematic Review of Pathomechanisms and Risk Factors for Calcific Uremic Arteriolopathy**

Cecilie Søndergaard, MD, Subagini Nagarajah, PhD, Jianlin Shen, PhD, Else-Marie Bladbjerg, PhD, Alexandra Scholze, PhD

**Search strings PubMed and EMBASE**

**Search string PubMed**

(Nephropathy OR kidney injury OR kidney OR renal dysfunction OR dialysis OR renal dialysis OR hemodialysis OR haemodialysis OR renal replacement therapy OR kidney replacement therapy OR CAPD OR APD OR CCPD OR renal insufficiency OR kidney insufficiency OR uremia OR uraemia OR uremic OR CKD OR CKF OR CRD OR CRF OR kidney disease OR kidney diseases OR kidney failure OR renal failure OR chronic renal insufficiency OR chronic kidney insufficiency OR ESRD OR ESKD OR ESRF OR ESKF OR end stage kidney disease OR end-stage kidney disease OR end stage renal disease OR end-stage renal disease OR chronic kidney failure OR chronic renal failure OR end stage kidney failure OR end-stage kidney failure OR end stage renal failure OR end-stage renal failure OR chronic kidney disease) AND (calciphylaxis OR calcific uremic arteriolopathy OR calcific uremic arteriopathy OR CUA) NOT (animals[MH] NOT humans[MH])

**Search string EMBASE**

\#	Query

1	Nephropathy.mp. or *kidney disease/

2	*chronic kidney failure/ or kidney injury.mp. or *kidney injury/

3	*kidney failure/ or *kidney dysfunction/ or renal dysfunction.mp.

4	*dialysis/ or dialysis.mp.

5	hemodialysis.mp. or *hemodialysis/

6	renal insufficiency.mp. or *kidney failure/

7	kidney insufficiency.mp. or *kidney failure/

8	uraemia.mp. or *uremia/

9	chronic renal insufficiency.mp.

10	chronic kidney disease.mp.

11	End stage renal disease.mp. or *chronic kidney failure/ or *end stage renal disease/

12	end stage kidney disease.mp.

13	Stage 5 kidney disease.mp.

14	1 or 2 or 3 or 4 or 5 or 6 or 7 or 8 or 9 or 10 or 11 or 12 or 13

15	calciphylaxis.mp.

16	calcific uremic arteriolopathy.mp.

17	calcific uremic arteriopathy.mp.

18	15 or 16 or 17

19	14 and 18

**Supplementary Table S1** Characteristics and quality rating (Good to Poor) of the reviewed studies.

| **Study, country*** | **Study design and study information** | **Quality** | **Calciphylaxis cases reported** | **Control group for the current review** | **Definition of CUA cases, as reported in the study** |
| --- | --- | --- | --- | --- | --- |
| Mehta 1990 [S1],  USA | Case-control study, investigates acquired protein C deficiency in CUA. Retrospective. | Fair | Dialysis patients with CUA  (n=5) | Dialysis patients without CUA  (n=12) | Diagnosis by radiographic evaluation. |
| Angelis 1997 [S2],  USA | Cross-sectional study,  investigates calciphylaxis prevalence. Retro-spective; 1995 to 1996. | Good | HD patients with CUA  (n=10) | HD patients without CUA  (n=232) | Clinical diagnosis. |
| Bleyer 1998 [S3],  USA | Case-control study,  investigates calciphylaxis risk factors and clinical presentation.  Retrospective; 1990 to 1996. | Good | ESKD patients with CUA  (n=9) | Hemodialysis patients without CUA  (n=347) | Diagnosis is based on clinical record and skin biopsy. |
| Zacharias 1999 [S4], Canada | Matched case-control study, investigates calcium use prior to CUA. Retrospective;  Over a 3-year period. | Good | ESKD with CUA  (n=8) | ESKD without CUA  (n=37) | Diagnosis based on clinical identification of hard subcutaneous plaques and bone scan detection of subcutaneous calcium deposits. |
| Ahmed 2001 [S5],  USA | Case-control study,  investigates phosphate and osteopontin in calciphylaxis, Retro-spective; 1995 to 2000. | Good | ESKD patients with CUA  (n=10) | ESKD patients without CUA  (n=180) | Diagnosis is based on skin biopsies and clinical picture consistent with CUA. |
| Mazhar 2001 [S6], USA | Matched cases-control study, investigates CUA risk factors and mortality. Retrospective; 1989 to 2000. | Fair to good | ESKD with CUA  HD (n=18);  Ntx (n=1) | Hemodialysis patients without CUA  (n=54) | Diagnosis based on skin lesions (painful/pruritic, subcutaneous nodules, ulceration/eschar formation), in 84% also a skin biopsy (small artery medial calcification and intimal hyperplasia, fat necrosis). |
| Wilmer 2001 [S7],  USA | Matched case-control study, investigates transcutaneous oxygen tension in CUA. Retrospective; 1994 to 1999. | Good | CUA cases: HD (n=14); PD (n=5); non-dialysis (n=2) | Controls without CUA: HD (n=13); PD (n=6); non-dialysis (n=2) | Diagnosis primarily based on clinical presentation and evidence of microcalcifications in the xeroradiography, with skin biopsies performed in some cases. |
| Fine 2002 [S8], Canada | Matched case-control study, investigates risk factors, treatment, and outcomes in calciphylaxis. | Good | Dialysis patients with CUA (n=36); subgroup: “plaques-only” CUA (n=24) | Dialysis patients no CUA  (n=1620)  subgroup analysis(n=48) | Diagnosis was based on clinical features (subcutaneous indurated plaques, ulcerations, positive bone scan, some patients had an additional X-ray of the legs or a skin biopsy). |
| Weenig 2007 [S9],  USA | Cross-sectional study,  Calciphylaxis risk factors and outcomes. Retrospective; 1992 to 2002. | Fair to good | CUA cases: HD (n=41); PD  (n=9), non-dialysis (n=15) | Dialysis patients without CUA, matched for age and sex (n=98) | Diagnosis based on clinical criteria, 86% biopsy confirmed (pannicular arterioles with medial calcification, intimal fibroplasia and thrombosis; cutaneous necrosis) |
| Cranenburg 2008 [S10], Germany, Nether-lands | Cross-sectional study, investigated cardiovascular calcification parameters. | Fair to good | HD with CUA (n=52) | HD without CUA (n=10) | Not specified. |
| Amuluru 2009 [S11],  USA | Case-control study,  investigates metal deposition in CUA. Retrospective; 2000 to 2007. | Fair | CKD patients with CUA  (n=12) | CKD patients without CUA  (n=5) | Diagnosis based on both clinical and histological (medial calcification, intimal hyperplasia, necrosis) CUA signs. |
| Hayashi 2012 [S12],  Japan | Nationwide survey and matched cases-control study, investigates CUA in ESKD. | Fair to good | HD patients with CUA  (n=28) | HD patients without CUA (n=56) | Diagnosis in line with Hayashi’s criteria. |
| Kramann 2013 [S13],  Germany | Case-control study, investigates CUA ectopic osteogenesis and matrix remodeling. Retro-spective; 2006 to 2012. | Fair to good | CKD/ESKD with CUA  (n=7) | Dialysis patients without CUA (n=7) | Diagnosis by clinical presentation and skin biopsies. |
| Nigwekar 2013 [S14],  USA | Matched cases-control study, investigates statin use in CUA. Retro-spective; 2002 to 2021. | Good | HD patients with CUA (n=62) | HD patients without CUA  (n=124) | All diagnosis were biopsy-confirmed (including von Kossa stains) by one or more dermatopathologists. |
| Smith 2013 [S15], Australia | Cross-sectional study, investigates fetuin-A and fetuin-A-containing CPPs. Retrospective; 2011 to 2012. | Good | HD with CUA  (n=6); PD (n=18); CKD non-dialysis (n=11); CID (n=13); healthy (n=24) | HD without CUA  (n=36) | Diagnosis was based on clinical criteria, partially biopsy-confirmed. |
| Floege 2015 [S16], multiple countries | RCT, investigates the impact of cinacalcet on CUA events; part of the EVOLVE trial. | Fair to good | HD patients with CUA (n=24) | HD patients without CUA (n=3837) | Diagnosis by local investigators, some were confirmed through biopsy or other methods. |
| Nigwekar 2016 [S17],  USA | Matched cases-control study, investigates CUA risk factors. Retro-spective; 2010 to 2014. | Good | HD with CUA (n=1030) | HD without CUA (n=2060) | Diagnosis based on skin biopsy confirmation in 55%, remaining cases were diagnosed clinically |
| Zhang 2016 [S18],  USA | Observational cohort, characterizes CUA in PD  Retrospective; 2001 to 2015. | Fair to good | PD with CUA  (n=7) | PD without CUA  (n=56) | Diagnosis was clinically suspected, and confirmed histopathologically in all patients. |
| Brandenburg 2017 [S19], Germany | Cross-sectional study,  investigates CUA characteristics derived from a nationwide registry (The German Calciphylaxis Registry); 2006 to 2015. | Fair to good | Calciphylaxis  (no or mild CKD (n=7); CKD non-HD (n=18); Ntx (n=10); PD (n=25); HD/HDF (n=193) | we used a HD control population, DOPPS practice monitor.  (<https://www.dopps.org/DPM-HD/Germany/>) accessed 03/2024 | Diagnosis based on clinical criteria; in 45% of cases also based on histological examination of a skin biopsy. |
| Chen 2017 [S20], USA | Cross-sectional study,  investigates dermatopathology in calciphylaxis. Retro-spective; 1999 to 2014. | Fair | CKD with CUA (n=45); CKD stage 5 with CUA (n=34) | CKD stage 5 without CUA  (n=26) | Diagnosis based on clinical criteria and skin biopsies to the level of the panniculus. |
| Nigwekar 2017 [S21],  USA | Matched case-control study, investigates carboxylation of matrix Gla protein in CUA; 2013 to 2014. | Good | HD patients with CUA  (n=20) | HD patients without CUA  (n=20) | Skin biopsy-confirmed CUA. |
| Rothe 2017 [S22],  Germany | Cross-sectional study, investigates SNPs in CUA. Retrospective; 2006 to 2014. | Good | ESKD with CUA: HD (n=128); non-HD (n=16) | ESKD without CUA, all dialysis  (n=370) | Diagnosis based on clinical criteria; in 45% of cases also based on histological examination of a skin biopsy. |
| Cassius 2018 [S23],  France | Case-control study,  investigates cutaneous calcifications. Retro-spective; 2012 to 2015. | Good | HD patients with CUA  (n=7) | HD patients without CUA  (n=17) | Diagnosis is based on clinical and histological criteria. |
| Dobry 2018 [S24],  USA | Matched cases-control study, investigates hypercoagulability in calciphylaxis. Retro-spective; 2006 to 2014. | Good | CKD stage 1-4 with CUA (n=10); CKD stage 5 with CUA (n=28) | CKD stage 5 without CUA (n=43) | Clinical diagnosis of calciphylaxis by a dermatologist. |
| Ellis 2018 [S25], USA | Case-control study, investigates histological findings in CUA. Retro-spective; 2014 to 2015. | Good | ESKD with suspected CUA (n=37) | ESKD patients without CUA  (n=34) | Suspicion of CUA based on medical record, not the pathology report. |
| Gaisne 2020 [S26],  France | A cohort and a matched case-control study, investigates CUA risk factors, treatments, and survival. Retro-spective; 2006 to 2016. | Good** | CUA cases:  CKD stage 4/5 non-dialysis (n=19);  HD (n=67);  PD (n=3) | HD without CUA  (n=140) | Diagnosis following Hayashi’s criteria. |
| Liu 2021 [S27],  China | Matched cases-control study, investigates calciphylaxis risk factors.  Retrospective; 2017 to 2018. | Good | HD patients newly diagnosed with CUA  (n=20) | HD patients without CUA  (n=40) | Diagnosis evaluation of skin lesions and skin biopsies. |
| Rapp 2021 [S28], Germany and Belgium | Data collected in 4 different patient cohorts; investigates hepatic and vascular vitamin K status. | Fair to good*** | HD with CUA  (n=32); aortic valve calcifica-tion (n=99); AF (n=30); general population (n=65) | HD without CUA  (n=40) | Cases from the German Calciphylaxis Registry, diagnosis based on clinical CUA signs and partially histological examination of a skin biopsy. |
| Liu 2022 [S29], China | Cross-sectional study, investigates calciphylaxis clinical characteristics and prevalence. Retro-spective; 2018 to 2019. | Good | HD patients with CUA  (n=48) | HD patients without CUA  (n=3819) | Diagnosis based on appearance of skin lesions, and skin biopsy (71%) and/or bone scintigraphy (67%). |
| Colboc 2023 [S30],  France | Case-control study, investigates elastic fibers and calcifications in CUA. Retrospective; 2006 to 2017. | Good | ESKD patients with CUA  (n=14) | ESKD patients without CUA  (n=12) | Diagnosis based on Hayashi’s criteria, including skin biopsy. |
| Gleue 2023 [S31],  USA | Case-control study, investigates systemic calcifications in calciphylaxis. | Fair | Decedents with CKD and CUA  (n=7) | Decedents with CKD without CUA (n=5) | Diagnosis based on clinical criteria, all cases presented with skin lesions. |
| Jang 2023 [S32], Canada | Matched cases-control study, investigates risk factors, incidence, out-comes in CUA. Retrospective; 2017 to 2020. | Good | HD patients newly diagnosisd with CUA  (n=40) | HD patients without CUA  (n=80) | Diagnosis based on electronic medical records. |
| Røndbjerg 2023 [S33],  Denmark | Cross-sectional study, investigates cutaneous vascular calcifications in CKD and CUA. | Good | ESKD and CUA (n=9); CKD stage 3b/4 (n=12); reference cases (n=6) | ESKD without CUA (n=12) | Current or previous clinical CUA diagnosis. |
| Toussaint 2024 [S34],  Australia and New Zealand | Observational cohort, investigates CUA incidence, outcomes and chracteristics. Retrospective; 2019 to 2022. | Fair to Good | KRT with CUA  (n=333) | KRT without CUA  (n=46526) | Diagnosis based on 'the opinion of the treating physician'. |
| Napoleon 2025 [S35], USA | Matched cases-control study, investigates molecular signalling in calciphylaxis skin. Prospective. | Good | ESKD patients with CUA (n=40) | ESKD patients without CUA (n=40) | Diagnosis based on skin lesions consistent with  Calciphylaxis. |

CID; Chronic Inflammatory Disease, CKD; Chronic Kidney Disease, CUA; Calcific Uremic Arteriolopathy, DOPPS; Dialysis Outcomes and Practice Patterns Study, ESKD; End-Stage Kidney Disease, HD; Hemodialysis, HDF; Hemodiafiltration, Ntx; Kidney Transplantation, PD; Peritoneal Dialysis

* Patients’ country of residence.

** Case-Control part assessed.

*** Cross-Sectional part assessed.

**Supplementary Table S2** Effect direction votes of potential risk factors, pathomechanisms, and related parameters compared between CUA and a suitable control population. Only parameters with data availability from all continents that had published such comparisons are shown.

| **Parameter** | **North America** | **Europe** | **Asia** | **Oceania** |
| --- | --- | --- | --- | --- |
| **Female**  (%, female to male ratio) | **Higher:** Ahmed 2001 [S5], Angelis 1997 [S2], Bleyer 1998 [S3], Fine 2002 [S8], Mazhar 2001 [S6], Wilmer 2001 [S7], Zacharias 1999 [S4], Zhang 2016 [S18] | **Higher:** Brandenburg 2017 [S19], Colboc 2023 [S30], Kramann 2013 [S13], Rothe 2017 [S22]  **Lower:** Røndbjerg 2023 [S33] | **Higher:** Hayashi 2012 [S12]  **Lower:** Liu 2021 [S27], Liu 2022 [S29] | **Higher:** Smith 2013 [S15], Toussaint 2024 [S34] |
| **BMI**  (BMI >30, obesity, BMI at time of CUA diagnosis, weight) | **Higher:** Ahmed 2001 [S5], Bleyer 1998 [S3], Jang 2023 [S32], Mazhar 2001 [S6], Nigwekar 2013 [S14], Nigwekar 2016 [S17], Weenig 2007 [S9], Wilmer 2001 [S7], Zacharias 1999 [S4], Zhang 2016 [S18]  **Lower:** Nigwekar 2017 [S21] | **Higher:** Cassius 2018 [S23], Colboc 2023 [S30], Gaisne 2020 [S26], Kramann 2013 [S13]  **Lower:** Røndbjerg 2023 [S33] | **Higher:** Liu 2021 [S27], Liu 2022 [S29] | **Higher:** Toussaint 2024 [S34] |
| **Age**  (at CUA diagnose) | **Higher:** Wilmer 2001 [S7]  **Equal:** Nigwekar 2013 [S14]  **Lower:** Ahmed 2001 [S5], Angelis 1997 [S2], Bleyer 1998 [S3], Fine 2002 [S8], Mazhar 2001 [S6], Zacharias 1999 [S4], Zhang 2016 [S18] | **Higher:** Brandenburg 2017 [S19], Colboc 2023 [S30], Røndbjerg 2023 [S33]  **Lower:** Kramann 2013 [S13] | **Lower:** Liu 2022 [S29] | **Higher:** Toussaint 2024 [S34]  **Equal:** Smith 2013 [S15] |
| **Calcium**  (total, serum, ionized; worst value within 6 months or 12-months average prior to CUA diagnosis, before CUA diagnose) | **Higher:** Angelis 1997 [S2]. Fine 2002 [S8]  **Equal:** Jang 2023 [S32], Weenig 2007 [S9]  **Lower:** Bleyer 1998 [S3], Mazhar 2001 [S6], Wilmer 2001 [S7], Zacharias 1999 [S4] | **Higher:** Colboc 2023 [S30], Gaisne 2020 [S26], Røndbjerg 2023 [S33]  **Equal:** Brandenburg 2017 [S19] | **Higher:** Liu 2021 [S27], Liu 2022 [S29] | **Higher:** Smith 2013 [S15] |
| **Phosphate**  (serum, worst value within 6 months or 12-months average prior to CUA diagnosis, before CUA diagnose) | **Higher:** Ahmed 2001 [S5], Angelis 1997 [S2], Bleyer 1998 [S3], Fine 2002 [S8], Mazhar 2001 [S6], Nigwekar 2013 [S14], Nigwekar 2016 [S17], Nigwekar 2017 [S21], Weenig 2007 [S9], Zacharias 1999 [S4], Zhang 2016 [S18]  **Lower:** Jang 2023 [S32], Wilmer 2001 [S7] | **Higher:** Colboc 2023 [S30], Gaisne 2020 [S26], Røndbjerg 2023 [S33]  **Lower:** Brandenburg 2017 [S19] | **Higher:** Liu 2021 [S27], Liu 2022 [S29]  **Equal:** Hayashi 2012 [S12] | **Lower:** Smith 2013 [S15] |
| **Ca x P**  (corrected, worst value within 6 months or 12-months average prior to CUA diagnosis, before CUA diagnosis) | **Higher:** Ahmed 2001 [S5], Angelis 1997 [S2], Bleyer 1998 [S3], Fine 2002 [S8], Mazhar 2001 [S6], Nigwekar 2013 [S14], Weenig 2007 [S9], Zacharias 1999 [S4] | **Higher:** Colboc 2023 [S30], Gaisne 2020 [S26] | **Higher:** Liu 2021 [S27] | **Lower:** Smith 2013 [S15] |
| **PTH**  (intact, whole, PTH value divided by the upper limit of normal range; serum, plasma; worst value within 6 months or 12-months average prior to CUA diagnose, before CUA diagnose) | **Higher:** Angelis 1997 [S2], Jang 2023 [S32], Mazhar 2001 [S6], Nigwekar 2013 [S14], Nigwekar 2016 [S17], Nigwekar 2017 [S21], Weenig 2007 [S9], Wilmer 2001 [S7], Zacharias 1999 [S4]  **Lower:** Ahmed 2001 [S5], Bleyer 1998 [S3], Fine 2002 [S8], Zhang 2016 [S18] | **Higher:** Gaisne 2020 [S26]  **Lower:** Brandenburg 2017 [S19], Røndbjerg 2023 [S33] | **Higher:** Hayashi 2012 [S12], Liu 2021 [S27], Liu 2022 [S29] | **Higher:** Smith 2013 [S15] |
| **Albumin**  (serum; worst value within 6 months or 12-months average prior to CUA diagnosis, before CUA diagnose) | **Lower:** Ahmed 2001 [S5], Bleyer 1998 [S3], Mazhar 2001 [S6], Nigwekar 2013 [S14], Nigwekar 2017 [S21], Zacharias 1999 [S4], Zhang 2016 [S18]  **Equal:** Nigwekar 2016 [S17], Weenig 2007 [S9] | **Lower:** Brandenburg 2017 [S19], Colboc 2023 [S30], Gaisne 2020 [S26], Røndbjerg 2023 [S33] | **Lower:** Hayashi 2012 [S12], Liu 2021 [S27], Liu 2022 [S29] | **Lower:** Smith 2013 [S15] |
| **Diabetes mellitus**  (prior to CUA diagnose, Diabetes mellitus type 2) | **Higher:** Bleyer 1998 [S3], Fine 2002 [S8], Jang 2023 [S32], Mazhar 2001 [S6], Nigwekar 2013 [S14], Nigwekar 2016 [S17], Weenig 2007 [S9], Wilmer 2001 [S7], Zhang 2016 [S18]  **Lower:** Nigwekar 2017 [S21] | **Higher:** Brandenburg 2017 [S19], Colboc 2023 [S30], Gaisne 2020 [S26]  **Equal:** Kramann 2013 [S13]  **Lower:** Røndbjerg 2023 [S33] | **Higher:** Liu 2021 [S27], Liu 2022 [S29] | **Higher:** Toussaint 2024 [S34] |
| **Cardiovascular disease**  (CAD, HF, chronic HF, PAD, stroke, AF, macro-/cerebro-vascular disease) | **Equal:** Nigwekar 2017 [S21] | **Higher:** Gaisne 2020 [S26]  **Lower:** Gaisne 2020 [S26], Røndbjerg 2023 [S33] | **Higher:** Liu 2021 [S27], Liu 2022 [S29]  **Equal:** Liu 2021 [S27] | **Higher:** Toussaint 2024 [S34] |

AF; Atrial Fibrillation, BMI; Body Mass Index, CAD; Coronary Artery Disease, Ca x P; Calcium-Phosphorus Product, CUA; Calcific Uremic Arteriolopathy, HF; Heart Failure, PAD; Peripheral Artery Disease, PTH; Parathyroid Hormone

**Supplementary Table S3** Comparison of dermatopathological findings between patients with calcific uremic arteriolopathy (CUA) and a suitable control population.

| **Study** | **Parameter** | **CUA group** | **Non-CUA group** |
| --- | --- | --- | --- |
| Cassius 2018 [S23] | **Cutaneous calcifications**  Cutaneous calcification characteristics | 86%  Hypodermic cutaneous calcifications only; 3/7 cases with interstitial and vessel calcification <500 μm; 2/7 cases had calcifications in medium-sized vascular bed only; 1/7 with interstitial calcification only. Vascular calcifications, found in 4/7 cases, were circumferential with thrombosis in 3/7 cases. | 18%  Cutaneous calcifications were diffuse in dermis and hypodermis and measured several mm. All findings were in accordance with metastatic calcinosis cutis. |
| Chen 2017 [S20] | **Thrombi** - None  - Focal  - Diffuse | 18%  32%  50% | 46%  27%  27% |
|  | **Calcification** - None  - Focal  - Diffuse | 21%  50%  29% | 92%  8%  0 |
|  | **Extravascular calcification** | 50% | 0 |
|  | **Perieccrine calcification** | 0 | 0 |
|  | **Superficial dermis calcium** | 3% | 0 |
|  | **Mid-dermal calcium** | 26% | 0 |
|  | **Superficial fat calcium** | 47% | 0 |
|  | **Deep panniculus calcium** | 4% | 18% |
|  | **Superficial dermis thrombi** | 32% | 8% |
|  | **Mid-dermal thrombi** | 29% | 0 |
|  | **Deep dermis thrombi** | 35% | 15% |
|  | **Superficial fat thrombi** | 47% | 27% |
|  | **Deep panniculus thrombi** | 21% | 31% |
|  | **Thrombosis in calcified vessel** | 15% | 0 |
|  | **Ulceration** | 21% | 8% |
|  | **Dermal angioplasia**  - None  - Focal  - Moderate  - Diffuse | 29%  12%  35%  24% | 88%  4%  0  8% |
| Colboc 2023 [S30] | **Interstitial calcification**  - Adipocytes membrane  - Dermic elastic fibers  - Sweat glands basal  lamina | 93%  79%  36% | 17%  25%  58% |
|  | **Vascular calcification**  - Voluminous, circum-  ferential  - Small-sized vessels  - Medium-sized vessels  - Thrombosis | 100%  100%  43%  21% | 0  50%  33%  0 |
|  | **Major elastic fiber dimension** | Comparable | |
|  | **Elastic fibers straightness** (eccentricity) | 0.92  (7/14 cases) | 0.88  (8/12 cases) |
|  | **Calcification ultrastructure** | Small calcic spherules measuring 500 nm to 1 μm could typically be shown. | |
|  |  | Micrometric structural organization:  spherules were regrouped into large plaques. | Micrometric structural organization: spherules were coalescent without plaque formation. |
| Ellis 2018 [S25] | **Small vessel lesions**  - Calcification  - Thrombosis  - Intimal hyperplasia  - Calcification + thrombosis  - Calcification + intimal  hyperplasia | 57%  38%  36%  5%  19%  0% | 32%  19%  14%  12%  5%  5% |
|  | **Extravascular soft tissue calcification**  - Perieccrine | 27%  5% | 40%  2% |
|  | **Vascular + extravascular calcification** | 8% | 9% |
|  | **Vascular or extravascular calcification** | 46% | 49% |
| Gleue 2023 [S31] | **Cutaneous and extracutaneous calcification characteristics** | Cutaneous: intimal calcification with thromboses 7/7 cases,  Extracutaneous manifestation 5/7 cases:  - Kidneys; 5/7 cases showed calcifications of tubules and small vessel  - 3/7 cases had calcifications and thromboses of small pancreatic vessels  - Other locations that showed calcifications of small vessels: spleen, adrenal gland, mesenterial vasculature, lungs | no findings of calciphylaxis  Extracutaneous manifestation 2/5 cases:  - Kidneys; tubular and medium-large vessel intimal calcifications  - Pancreas; medium-large vessel intimal calcifications |
| Kramann 2013 [S13] | **Calcified area in skin**  (von Kossa; semiquantitative evaluation) | 3.57±0.35 | 0.07±0.19 |
| Amuluru 2009 [S11] | **Skin metal deposition**  Tissue iron (μg/g)ˣ  Tissue aluminum (μg/g)ˣˣ  Tissue gadolinium (μg/g)ˣˣˣ | 889.3 SD 361.5  1493.3 SD 1960.1  45.7 SD 87.3¨ (n=8) | 280.4 SD 139.5  99.2 SD 45.5  0.4 SD 0.3¨ |

ˣ= iron medication exposure: at least 4 CUA cases received oral or parenteral iron supplement, controls not reported.

ˣˣ= aluminium-based phosphate binder exposure: CUA cases not exposed, controls not reported.

ˣˣˣ= gadolinium contrast exposure: 8 CUA cases exposed, controls all exposed.

¨= value was calculated by us

**Supplementary references**

S1 Mehta RL, Scott G, Sloand JA, Francis CW. Skin necrosis associated with acquired protein C deficiency in patients with renal failure and calciphylaxis. Am J Med. 1990 Mar;88(3):252-7

S2 Angelis M, Wong LL, Myers SA, Wong LM. Calciphylaxis in patients on hemodialysis: a prevalence study. Surgery. 1997 Dec;122(6):1083-9; discussion 1089-90

S3 Bleyer AJ, Choi M, Igwemezie B, de la Torre E, White WL. A case control study of proximal calciphylaxis. Am J Kidney Dis. 1998 Sep;32(3):376-83

S4 Zacharias JM, Fontaine B, Fine A. Calcium use increases risk of calciphylaxis: a case-control study. Perit Dial Int. 1999 May-Jun;19(3):248-52

S5 Ahmed S, O'Neill KD, Hood AF, Evan AP, Moe SM. Calciphylaxis is associated with hyperphosphatemia and increased osteopontin expression by vascular smooth muscle cells. Am J Kidney Dis. 2001 Jun;37(6):1267-76

S6 Mazhar AR, Johnson RJ, Gillen D, Stivelman JC, Ryan MJ, Davis CL, Stehman-Breen CO. Risk factors and mortality associated with calciphylaxis in end-stage renal disease. Kidney Int. 2001 Jul;60(1):324-32

S7 Wilmer WA, Voroshilova O, Singh I, Middendorf DF, Cosio FG. Transcutaneous oxygen tension in patients with calciphylaxis. Am J Kidney Dis. 2001 Apr;37(4):797-806

S8 Fine A, Zacharias J. Calciphylaxis is usually non-ulcerating: risk factors, outcome and therapy. Kidney Int. 2002 Jun;61(6):2210-7

S9 Weenig RH, Sewell LD, Davis MD, McCarthy JT, Pittelkow MR. Calciphylaxis: natural history, risk factor analysis, and outcome. J Am Acad Dermatol. 2007 Apr;56(4):569-79. doi: 10.1016/j.jaad.2006.08.065

S10 Cranenburg EC, Vermeer C, Koos R, Boumans ML, Hackeng TM, Bouwman FG, Kwaijtaal M, Brandenburg VM, Ketteler M, Schurgers LJ. The circulating inactive form of matrix Gla Protein (ucMGP) as a biomarker for cardiovascular calcification. J Vasc Res. 2008;45(5):427-36

S11 Amuluru L, High W, Hiatt KM, et al. Metal deposition in calcific uremic arteriolopathy. J Am Acad Dermatol. 2009;61:73-79

S12 Hayashi M, Takamatsu I, Kanno Y, Yoshida T, Abe T, Sato Y; Japanese Calciphylaxis Study Group. A case-control study of calciphylaxis in Japanese end-stage renal disease patients. Nephrol Dial Transplant. 2012 Apr;27(4):1580-4

S13 Kramann R, Brandenburg VM, Schurgers LJ, Ketteler M, Westphal S, Leisten I, Bovi M, Jahnen-Dechent W, Knüchel R, Floege J, Schneider RK. Novel insights into osteogenesis and matrix remodelling associated with calcific uraemic arteriolopathy. Nephrol Dial Transplant. 2013 Apr;28(4):856-68

S14 Nigwekar SU, Bhan I, Turchin A, Skentzos SC, Hajhosseiny R, Steele D, Nazarian RM, Wenger J, Parikh S, Karumanchi A, Thadhani R. Statin use and calcific uremic arteriolopathy: a matched case-control study. Am J Nephrol. 2013;37(4):325-32

S15 Smith ER, Cai MM, McMahon LP, Pedagogos E, Toussaint ND, Brumby C, Holt SG. Serum fetuin-A concentration and fetuin-A-containing calciprotein particles in patients with chronic inflammatory disease and renal failure. Nephrology (Carlton). 2013 Mar;18(3):215-21

S16 Floege J, Kubo Y, Floege A, Chertow GM, Parfrey PS. The Effect of Cinacalcet on Calcific Uremic Arteriolopathy Events in Patients Receiving Hemodialysis: The EVOLVE Trial. Clin J Am Soc Nephrol. 2015 May 7;10(5):800-7

S17 Nigwekar SU, Zhao S, Wenger J, Hymes JL, Maddux FW, Thadhani RI, Chan KE. A Nationally Representative Study of Calcific Uremic Arteriolopathy Risk Factors. J Am Soc Nephrol. 2016 Nov;27(11):3421-3429

S18 Zhang Y, Corapi KM, Luongo M, Thadhani R, Nigwekar SU. Calciphylaxis in peritoneal dialysis patients: a single center cohort study. Int J Nephrol Renovasc Dis. 2016 Sep 19;9:235-241

S19 Brandenburg VM, Kramann R, Rothe H, Kaesler N, Korbiel J, Specht P, Schmitz S, Krüger T, Floege J, Ketteler M. Calcific uraemic arteriolopathy (calciphylaxis): data from a large nationwide registry. Nephrol Dial Transplant. 2017 Jan 1;32(1):126-132

S20 Chen TY, Lehman JS, Gibson LE, Lohse CM, El-Azhary RA. Histopathology of Calciphylaxis: Cohort Study With Clinical Correlations. Am J Dermatopathol. 2017 Nov;39(11):795-802

S21 Nigwekar SU, Bloch DB, Nazarian RM, Vermeer C, Booth SL, Xu D, Thadhani RI, Malhotra R. Vitamin K-Dependent Carboxylation of Matrix Gla Protein Influences the Risk of Calciphylaxis. J Am Soc Nephrol. 2017 Jun;28(6):1717-1722

S22 Rothe H, Brandenburg V, Haun M, Kollerits B, Kronenberg F, Ketteler M, Wanner C. Ecto-5' -Nucleotidase CD73 (NT5E), vitamin D receptor and FGF23 gene polymorphisms may play a role in the development of calcific uremic arteriolopathy in dialysis patients - Data from the German Calciphylaxis Registry. PLoS One. 2017 Feb 17;12(2):e0172407

S23 Cassius C, Moguelet P, Monfort JB, Fessi H, Michel PA, Boulahia G, Cury K, Frances C, Senet P. Calciphylaxis in haemodialysed patients: diagnostic value of calcifications in cutaneous biopsy. Br J Dermatol. 2018 Jan;178(1):292-293

S24 Dobry AS, Ko LN, St John J, Sloan JM, Nigwekar S, Kroshinsky D. Association Between Hypercoagulable Conditions and Calciphylaxis in Patients With Renal Disease: A Case-Control Study. JAMA Dermatol. 2018 Feb 1;154(2):182-187

S25 Ellis CL, O'Neill WC. Questionable specificity of histologic findings in calcific uremic arteriolopathy. Kidney Int. 2018 Aug;94(2):390-395

S26 Gaisne R, Péré M, Menoyo V, Hourmant M, Larmet-Burgeot D. Calciphylaxis epidemiology, risk factors, treatment and survival among French chronic kidney disease patients: a case-control study. BMC Nephrol. 2020 Feb 26;21(1):63

S27 Liu Y, Zhang X, Xie X, Yang X, Liu H, Tang R, Liu B. Risk factors for calciphylaxis in Chinese hemodialysis patients: a matched case-control study. Ren Fail. 2021 Dec;43(1):406-416

S28 Rapp N, Brandenburg VM, Kaesler N, Bakker SJL, Stöhr R, Schuh A, Evenepoel P, Schurgers LJ. Hepatic and Vascular Vitamin K Status in Patients with High Cardiovascular Risk. Nutrients. 2021 Oct 1;13(10):3490

S29 Liu Y, Yang C, Yang X, Xie X, Liu H, Zhang L, Ye J, Jiang D, Zhang X, Liu B. Prevalence and Clinical Characteristics of Calciphylaxis in Chinese Hemodialysis Patients. Front Med (Lausanne). 2022 Jun 10;9:902171

S30 Colboc H, Moguelet P, Bazin D, Letavernier E, Sun C, Chessel A, Carvalho P, Lok C, Dillies AS, Chaby G, Maillard H, Kottler D, Goujon E, Jurus C, Panaye M, Tang E, Courville P, Boury A, Monfort JB, Chasset F, Senet P, Schanne-Klein MC. Elastic fiber alterations and calcifications in calcific uremic arteriolopathy. Sci Rep. 2023 Sep 19;13(1):15519

S31 Gleue C, Bois M, Guo R. Calciphylaxis in autopsy: A retrospective case-controlled study of 7 decedents. JAAD Case Rep. 2023 Mar 8;35:60-62

S32 Jang A, Mark Ho MH, Yan L. Characterization of Risk Factors for Calciphylaxis in Hemodialysis Patients in the Fraser Health Renal Program - A Matched Case-Control Retrospective Review. J Pharm Pract. 2023 Dec;36(6):1424-1437

S33 Røndbjerg AK, Gyldenløve M, Krustrup D, Rix M, Vejborg I, Lonn L, Jørgensen NR, Pasch A, Skov L, Hansen D. Cutaneous vascular calcifications in patients with chronic kidney disease and calcific uremic arteriolopathy: a cross-sectional study. J Nephrol. 2023 Sep;36(7):1991-1999. doi: 10.1007/s40620-023-01707-8

S34 Toussaint ND, Davies CE, Bongetti E, Ruderman I, Elder GJ, Hawley CM, Krishnasamy R, Roberts MA, Jardine MJ, De Zoysa JR, McDonald SP. Calciphylaxis Episodes in the Australia and New Zealand Dialysis and Transplant Registry. Kidney Int Rep. 2024 Jan 18;9(4):951-959

S35 Napoleon MA, Yang X, Zhang Y, Morrissey A, Krinsky S, Lazowski A, Bouchouari H, Brahim AM, Sellinger I, Edwards T, Jose A, Lotfolhzadeh S, Almiron R, Yin W, Siracuse JJ, Francis J, Lokau J, Garbers C, Del Carmen PM, Kolachalama VB, Dries R, Nazarian RM, Nigwekar SU, Chitalia VC. Activation and targetability of TYMP-IL-6-TF signaling in the skin microenvironment in uremic calciphylaxis. Sci Transl Med. 2025 Apr 23;17(795):eadn5772. doi: 10.1126/scitranslmed.adn5772. Epub 2025 Apr 23. PMID: 40267216

| **Section and Topic** | **Item #** | **Checklist item** | **Location where item is reported** |
| --- | --- | --- | --- |
| **TITLE** | | |  |
| Title | 1 | Identify the report as a systematic review. | P1 |
| **ABSTRACT** | | |  |
| Abstract | 2 | See the PRISMA 2020 for Abstracts checklist. | P2 |
| **INTRODUCTION** | | |  |
| Rationale | 3 | Describe the rationale for the review in the context of existing knowledge. | P3 |
| Objectives | 4 | Provide an explicit statement of the objective(s) or question(s) the review addresses. | P3 |
| **METHODS** | | |  |
| Eligibility criteria | 5 | Specify the inclusion and exclusion criteria for the review and how studies were grouped for the syntheses. | P4 |
| Information sources | 6 | Specify all databases, registers, websites, organisations, reference lists and other sources searched or consulted to identify studies. Specify the date when each source was last searched or consulted. | P4 |
| Search strategy | 7 | Present the full search strategies for all databases, registers and websites, including any filters and limits used. | P4,5; Supplementary material |
| Selection process | 8 | Specify the methods used to decide whether a study met the inclusion criteria of the review, including how many reviewers screened each record and each report retrieved, whether they worked independently, and if applicable, details of automation tools used in the process. | P4,5 |
| Data collection process | 9 | Specify the methods used to collect data from reports, including how many reviewers collected data from each report, whether they worked independently, any processes for obtaining or confirming data from study investigators, and if applicable, details of automation tools used in the process. | P4-6 |
| Data items | 10a | List and define all outcomes for which data were sought. Specify whether all results that were compatible with each outcome domain in each study were sought (e.g. for all measures, time points, analyses), and if not, the methods used to decide which results to collect. | P4-6 |
|  | 10b | List and define all other variables for which data were sought (e.g. participant and intervention characteristics, funding sources). Describe any assumptions made about any missing or unclear information. | n.a. |
| Study risk of bias assessment | 11 | Specify the methods used to assess risk of bias in the included studies, including details of the tool(s) used, how many reviewers assessed each study and whether they worked independently, and if applicable, details of automation tools used in the process. | P5 |
| Effect measures | 12 | Specify for each outcome the effect measure(s) (e.g. risk ratio, mean difference) used in the synthesis or presentation of results. | P5,6 |
| Synthesis methods | 13a | Describe the processes used to decide which studies were eligible for each synthesis (e.g. tabulating the study intervention characteristics and comparing against the planned groups for each synthesis (item #5)). | P5,6 |
|  | 13b | Describe any methods required to prepare the data for presentation or synthesis, such as handling of missing summary statistics, or data conversions. | P5,6 |
|  | 13c | Describe any methods used to tabulate or visually display results of individual studies and syntheses. | P5,6 |
|  | 13d | Describe any methods used to synthesize results and provide a rationale for the choice(s). If meta-analysis was performed, describe the model(s), method(s) to identify the presence and extent of statistical heterogeneity, and software package(s) used. | P5,6 |
|  | 13e | Describe any methods used to explore possible causes of heterogeneity among study results (e.g. subgroup analysis, meta-regression). | n.a. |
|  | 13f | Describe any sensitivity analyses conducted to assess robustness of the synthesized results. | n.a. |
| Reporting bias assessment | 14 | Describe any methods used to assess risk of bias due to missing results in a synthesis (arising from reporting biases). | n.a. |
| Certainty assessment | 15 | Describe any methods used to assess certainty (or confidence) in the body of evidence for an outcome. | P5 |
| **RESULTS** | | |  |
| Study selection | 16a | Describe the results of the search and selection process, from the number of records identified in the search to the number of studies included in the review, ideally using a flow diagram. | P6-8 |
|  | 16b | Cite studies that might appear to meet the inclusion criteria, but which were excluded, and explain why they were excluded. | none |
| Study characteristics | 17 | Cite each included study and present its characteristics. | Suppl. Table 1 |
| Risk of bias in studies | 18 | Present assessments of risk of bias for each included study. | Suppl. Table 1 |
| Results of individual studies | 19 | For all outcomes, present, for each study: (a) summary statistics for each group (where appropriate) and (b) an effect estimate and its precision (e.g. confidence/credible interval), ideally using structured tables or plots. | Figure 2 to 5 |
| Results of syntheses | 20a | For each synthesis, briefly summarise the characteristics and risk of bias among contributing studies. | P 8-15 |
|  | 20b | Present results of all statistical syntheses conducted. If meta-analysis was done, present for each the summary estimate and its precision (e.g. confidence/credible interval) and measures of statistical heterogeneity. If comparing groups, describe the direction of the effect. | Figure 2 to 5 |
|  | 20c | Present results of all investigations of possible causes of heterogeneity among study results. | n.a. |
|  | 20d | Present results of all sensitivity analyses conducted to assess the robustness of the synthesized results. | n.a. |
| Reporting biases | 21 | Present assessments of risk of bias due to missing results (arising from reporting biases) for each synthesis assessed. | n.a. |
| Certainty of evidence | 22 | Present assessments of certainty (or confidence) in the body of evidence for each outcome assessed. | P 8-15 |
| **DISCUSSION** | | |  |
| Discussion | 23a | Provide a general interpretation of the results in the context of other evidence. | P16 |
|  | 23b | Discuss any limitations of the evidence included in the review. | P20,21 |
|  | 23c | Discuss any limitations of the review processes used. | P20,21 |
|  | 23d | Discuss implications of the results for practice, policy, and future research. | P20,21 |
| **OTHER INFORMATION** | | |  |
| Registration and protocol | 24a | Provide registration information for the review, including register name and registration number, or state that the review was not registered. | P4 |
|  | 24b | Indicate where the review protocol can be accessed, or state that a protocol was not prepared. | P4 |
|  | 24c | Describe and explain any amendments to information provided at registration or in the protocol. | none |
| Support | 25 | Describe sources of financial or non-financial support for the review, and the role of the funders or sponsors in the review. | P22 |
| Competing interests | 26 | Declare any competing interests of review authors. | P22 |
| Availability of data, code and other materials | 27 | Report which of the following are publicly available and where they can be found: template data collection forms; data extracted from included studies; data used for all analyses; analytic code; any other materials used in the review. | Supplementary excel file, Open Science Framework (OSF): at https://doi.org/10.17605/OSF.IO/MC36K |
